# A ruthenium anticancer compound interacts with histones and impacts differently on epigenetic and death pathways compared to cisplatin

**DOI:** 10.18632/oncotarget.13711

**Published:** 2016-11-30

**Authors:** Cynthia Licona, Marie-Elodie Spaety, Antonelle Capuozzo, Moussa Ali, Rita Santamaria, Olivier Armant, Francois Delalande, Alain Van Dorsselaer, Sarah Cianferani, John Spencer, Michel Pfeffer, Georg Mellitzer, Christian Gaiddon

**Affiliations:** ^1^ INSERM 1113, Molecular Signaling of the Cell Stress Response and Pathology, Université de Strasbourg, Section Oncologie FMTS, Strasbourg, France; ^2^ Karlsruhe Institute of Technology (KIT), Institute of Toxicology and Genetics (ITG), Germany; ^3^ Department of Chemistry, School of Life Sciences, University of Sussex, Falmer, Brighton, East Sussex, UK; ^4^ Institut of Chemistry, UMR7177 CNRS, Université de Strasbourg, Laboratory of Metal-Induced Synthesis, France; ^5^ Institut Pluridisciplinaire Hubert Curien, Département Sciences Analytiques, Université de Strasbourg, France; ^6^ Department of Pharmacy, University of Naples Federico II, Naples, Italy

**Keywords:** epigenetics, ruthenium, p53, ER stress, cisplatin

## Abstract

Ruthenium complexes are considered as potential replacements for platinum compounds in oncotherapy. Their clinical development is handicapped by a lack of consensus on their mode of action. In this study, we identify three histones (H3.1, H2A, H2B) as possible targets for an anticancer redox organoruthenium compound (RDC11). Using purified histones, we confirmed an interaction between the ruthenium complex and histones that impacted on histone complex formation. A comparative study of the ruthenium complex versus cisplatin showed differential epigenetic modifications on histone H3 that correlated with differential expression of histone deacetylase (HDAC) genes. We then characterized the impact of these epigenetic modifications on signaling pathways employing a transcriptomic approach. Clustering analyses showed gene expression signatures specific for cisplatin (42%) and for the ruthenium complex (30%). Signaling pathway analyses pointed to specificities distinguishing the ruthenium complex from cisplatin. For instance, cisplatin triggered preferentially p53 and folate biosynthesis while the ruthenium complex induced endoplasmic reticulum stress and trans-sulfuration pathways. To further understand the role of HDACs in these regulations, we used suberanilohydroxamic acid (SAHA) and showed that it synergized with cisplatin cytotoxicity while antagonizing the ruthenium complex activity. This study provides critical information for the characterization of signaling pathways differentiating both compounds, in particular, by the identification of a non-DNA direct target for an organoruthenium complex.

## INTRODUCTION

Transition metal complexes, including those of ruthenium, have been under investigation for several years as scaffolds for generating novel molecules harboring anticancer properties. These metals present interesting properties that confer advantages for designing cytotoxic compounds such as: enabling, otherwise inaccessible to carbon-based chemistry, an octahedral geometry, a wide variety of redox potentials, accessibility of numerous oxidation states (I to IV) and interesting ligand exchange rates, enabling covalent interactions with biological macromolecules [1, 2]. In addition, ruthenium is hypothesized to be less toxic than platinum as it might be eliminated by iron metabolism mechanisms. A multitude of ligands have been used to produce various ruthenium complexes in the redox state (II) or (III). Most compounds are generated through complexation through a nitrogen atom of the ligand. Alternatively, organo-ruthenium compounds have been generated containing a covalent Ru-C bond (C, carbon) in the ligand. *In vitro* and *in vivo* biological studies established that several of these ruthenium-based compounds show high cytotoxicity towards a wide range of cancer cells and reduced side effects [1–12]. Gratifyingly, ruthenium-based complexes are not affected by platinum-induced resistance mechanisms. Based on these characteristics, two ruthenium-based complexes, NAMI-A and KP1019, have been tested in phase I and II clinical trials [13, 14]. However, the lack of success of ruthenium compounds in late stage clinical trials may reside in part in the relative lack of understanding of their exact mode of action and the important chemical determinants involved.

In this respect, the mechanism of action of ruthenium-based complexes remains a matter of debate. Several modes of action have been proposed, which include interaction with DNA and activation of DNA damage pathways [15–19], kinase inhibition [20] or other enzymatic activities [21, 22], including extracellular metallo-proteases [23], thioredoxin and cathepsin B [24] [25]. This variability may be due to differences in the structure of the ruthenium complexes, due to variations of the nature of the ligands as well as the type of bond linking the ligand to the ruthenium atom. In addition, no global approaches have been described so far that would give a more exhaustive and comprehensive understanding of the signaling pathways that are triggered in response to ruthenium-based compounds.

In this study, we have analyzed direct protein targets of RDC11 and changes in gene expression induced by this complex in comparison to the well-established anticancer metal-based drug cisplatin. RDC11 is an organo-ruthenium compound in which two acetonitriles, one phenanthroline, and one 2-phenylpyridine ligand are linked to the metal. The 2-phenylpyridine is cyclometalated to the ruthenium, i.e. it is bound to Ru via the nitrogen's lone pair and an ortho carbon atom of the phenyl unit. We previously demonstrated that RDC11 is highly cytotoxic (IC_50_ between 1–5 μM) on multiple cell lines including cisplatin resistant cells [18, 26]. Importantly, RDC11 reduces tumor growth in different models, including mouse syngeneic models (melanoma, lung cancer) and human xenografted models (glioma and ovarian cancer), with reduced toxicity towards healthy tissues compared to cisplatin [26, 27]. We have also previously shown that RDC11 and related compounds such as RDC34 induce p53-dependent and Endoplasmic Reticulum (ER) stress pathways. However, we also showed that both pathways could not account for all the biological effect of RDC11-related compounds [18, 27, 28]. Finally, structure activity studies have indicated that RDC11 and RDC34's cytotoxicity is at least partly related to their redox potential and the production of reactive oxygen species [26, 29].

To understand the mode of action of RDC11 we have used a proteomic approach identifying histones as potential proteins targeted by RDC11 and we have established the impact on the cellular transcriptome to identify novel signaling pathways that could elucidate the biological activity of RDC11. In addition, we have performed a comparative analysis with cisplatin in order to characterize specific signatures or similarities between cisplatin and our organoruthenium compound.

## RESULTS

### The cytotoxic organoruthenium complex RDC11 interacts with histones in cancer cells

**Figure 1:**
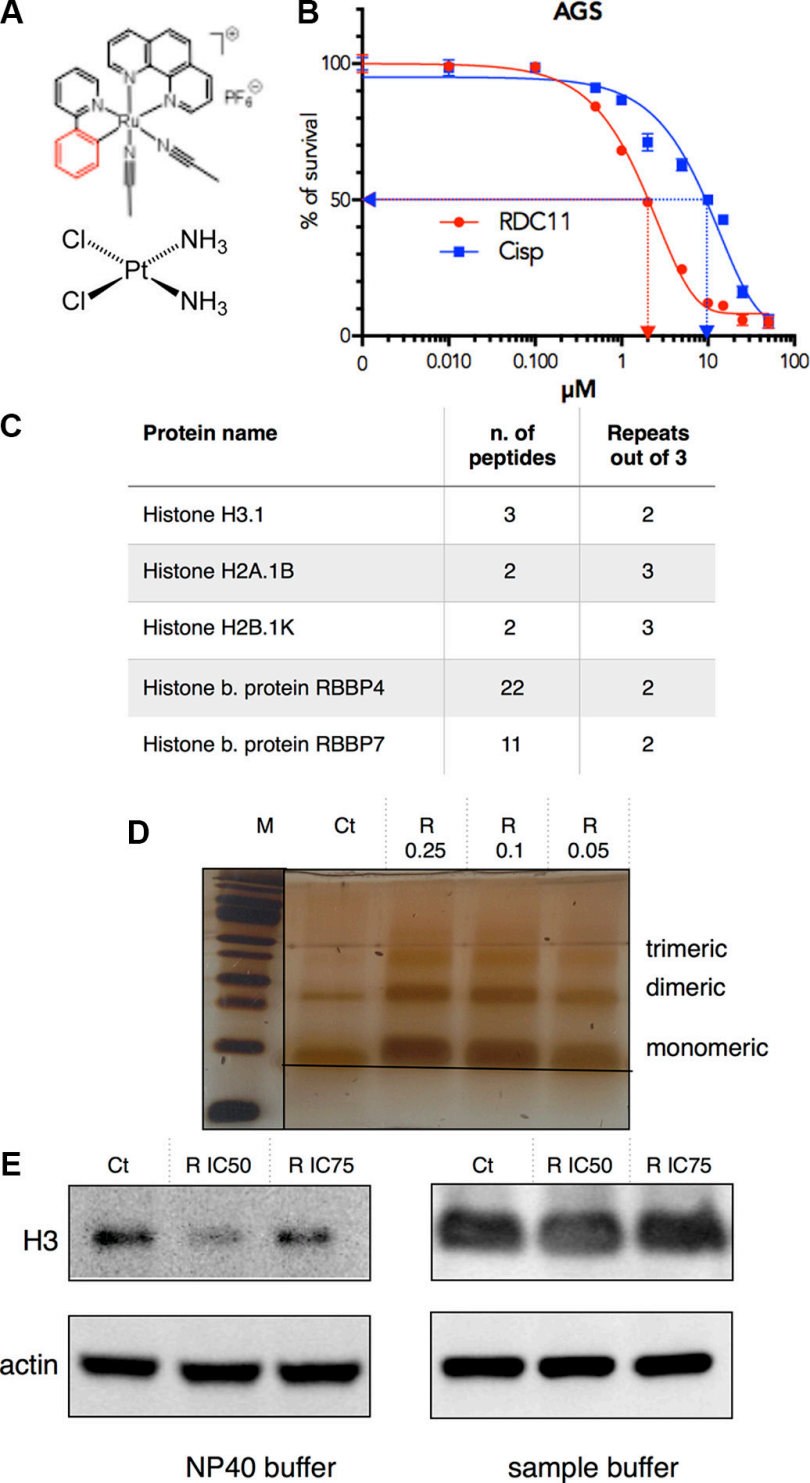
(**A**) Schematic representation of cisplatin and RDC11. (**B**) Survival curve of cancer cells AGS treated with cisplatin or RDC11. Cells were treated for 48 hours in 96-well plates and their survival was evaluated by MTT assay (*n* = 8). (**C**) Table indicating the number of peptides of histones and histone related proteins present in the RDC11 affinity chromatography and mass spectrometric analysis of cell extracts. Repeats are the number of experiment with presence of the peptides out of three experiments done. (**D**) Migration of histone H3-RDC11 complex on non-denaturing SDS-Page. 100 ng of purified histone H3 was incubated with increased concentrations of RDC11 (R 0.05 to 0.25 μM) for 1 hour. M is the molecular marker. Image is a silver stained gel of the complex showing the monomeric, dimeric and trimeric forms. (**E**) Proteins were extracted with the indicated buffer (NP40 or sample buffer) from AGS cells treated with RDC11 at the IC50 and the IC75 for 6 hours. Western blot analysis revealed histone H3 and actin protein levels.

We previously showed that, although RDC11 interacts with DNA, this does not fully explain its anticancer activity [26, 27, 30]. To identify RDC11's putative protein targets we used an affinity chromatography approach in which RDC11 was covalently bound to a solid matrix (HypoGel 400-COOH) (Supplementary Figure S5). As a source of possible protein targets we used cell extracts of gastric cancer AGS cells. AGS cells are more sensitive to RDC11 than to cisplatin (Figure 1A, 1B). Before loading, AGS cell extracts were treated with DNAse to maximize the liberation of DNA bound proteins. After incubation of the AGS cell extract for 1 h with the RDC11-matrix, the matrix was washed several times with buffer of increasing salt concentration and then proteins were eluted with a solution of free RDC11. Eluted proteins were identified using mass-spectrometry. The experiment was repeated several times and the results were each time compared to proteins identified using only the naked matrix. Three histones, H3.1, H2A.1B and H2B.1K, and histone binding proteins, RBBP4 and RBBP7, were found repeatedly to bind to the RDC11-matrix (Figure 1C). Despite their small molecular weight, for each histone, several peptides were identified by mass-spectrometry. As histones are DNA binding proteins and we previously showed that RDC11 binds to DNA, we decided to confirm that DNA did not mediate the interaction between RDC11 and histone. To do so, we used purified histones and incubated them with increasing amounts of RDC11. The mixture was then put to migration in a SDS-page under non-denaturing conditions (Figure 1D). Purified histones can migrate in non-denaturing gels as monomer, dimer and trimers. In the presence of RDC11, histone H3 migrated slower and with a more diffuse pattern suggesting the presence of histones of higher molecular weight due to the binding of RDC11. Changes in histone migration were observed with histone monomers, dimers and trimers. The interaction of the ruthenium complex with histone was confirmed using histone immunoprecipitation experiments followed by quantification of ruthenium in the precipitates be ICP-MS (Supplementary data 3C).

In cells, histones are the subject of multiple modifications that modulate their association with DNA, DNA compaction and also their translocation into the cytoplasm [31]. To assess the impact of RDC11-histone interactions in cells, we treated gastric cancer cells with RDC11 and extracted histones using two different protocols that allow either a preferential extraction of a soluble histone fraction (NP40 extraction buffer) or a full extraction of the histones bound to DNA (sample buffer) (Figure 1E). We observed that already, after three hours of treatment with RDC11 at the IC_50_ or the IC_75_ values, the soluble fraction of histone H3 was significantly reduced. Altogether these results indicated that RDC11 can bind directly to histones and that this binding can impact on histone function in cancer cells.

### Differential impact of RDC11 and cisplatin on histone modifications and epigenetic processes

**Figure 2:**
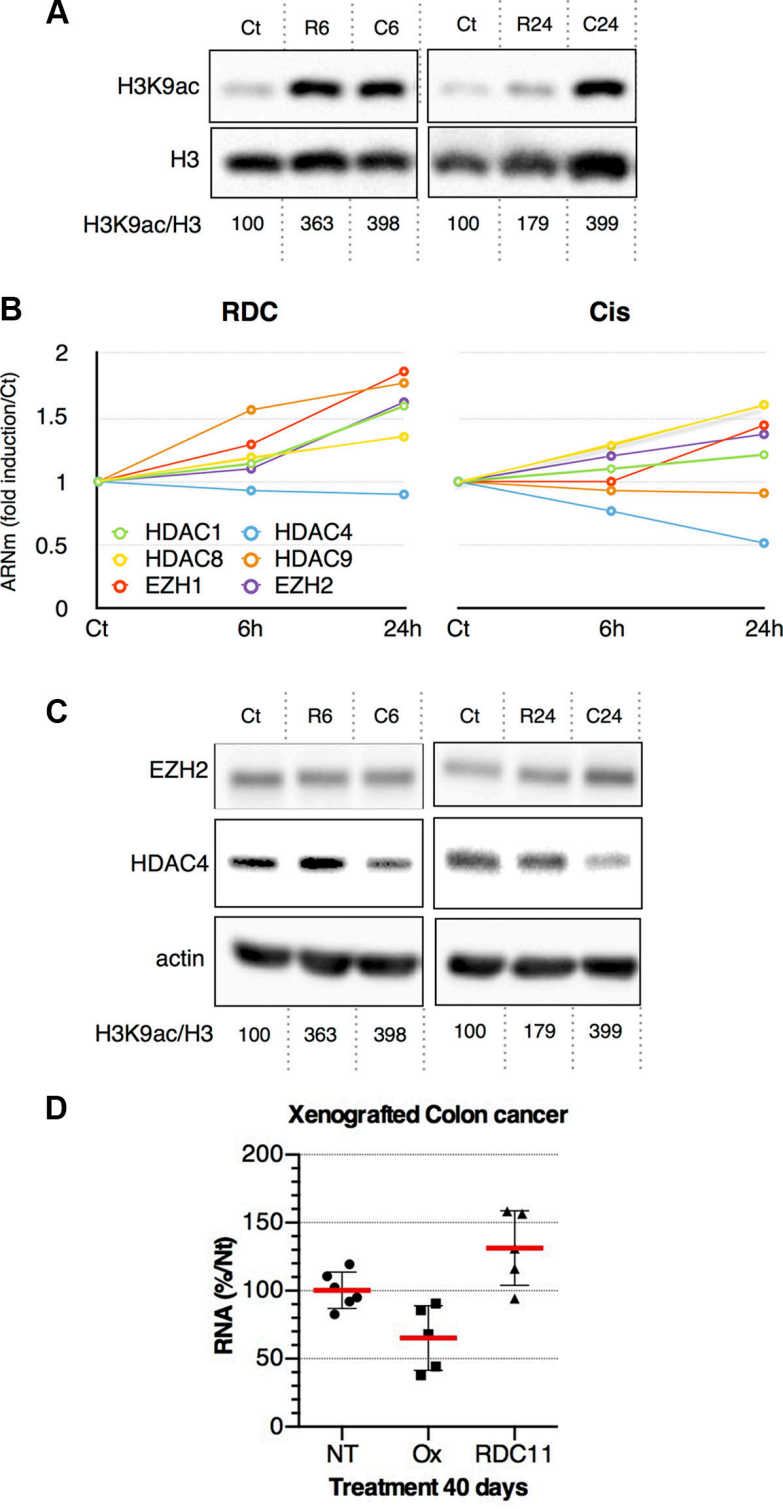
(**A**) Proteins were extracted from AGS treated with RDC11 (R6, R24) or cisplatin (C6, C24) for the indicated time (6 h, R6 and C6; 24 h, R24 and C24) using sample buffer. Western blot analysis revealed histone H3 acetyl lysine 7 (H3AK7), histone H3 (H3) and actin expression. Actin blot are shown in panel C. Quantifications of H3AK7/H3 are indicated below as measured by Pixi imager. (**B**) Curves are fold induction versus the control (Ct) for selected histone-modifying enzymes in RDC11 and cisplatin conditions. mRNA levels were assayed in AGS gastric cancer cells by RT-qPCR. Curves are means of fold induction versus the control (Ct) with SD (*n* = 3). *: *p* < 0.01. (**C**) Proteins were extracted from AGS treated with RDC11 (R6, R24) or cisplatin (C6, C24) for the indicated time. Western blot analysis revealed EZH2, HDAC4, and actin expression. (**D**) mRNA levels of *HDAC4* were assayed by RT-qPCR in fragments of human colon cancer xenografted in nude mice and after the treatment with cisplatin or RDC11. Graphs are means of fold induction versus the control (Ct) with SD (*n* = 5). *:*p* < 0.01.

As RDC11 binds to histones and diminishes the more soluble fraction of histone we hypothesized that it might impact on the post-translational modifications of histones. We analyzed, by Western-blot, the acetylation of histone H3 at lysine 9. We observed that histone H3 acetylation was strongly increased by cisplatin and RDC11 already 6 hours after treatment. However, after 24 hours there was a significant decrease of histone H3 acetylation under the RDC11 conditions while it remained elevated with cisplatin (Figure 2A).

To further understand the impact of RDC11 and cisplatin on epigenetic regulations related to histones, we analyzed the expression of several genes encoding for enzymes involved in histone modifications. RT-qPCR experiments indicated that RDC11 and cisplatin regulated differently the expression of several histone deacetylases (HDAC) or other enzymes such as EZH2 (Figure 2B). In particular, the expression of HDAC4 was repressed by cisplatin and not affected by RDC11 while EZH2 was induced. In addition and conversely to HDAC4, HDAC9 was induced by RDC11 and not by cisplatin. At the protein level, RDC11 induced HDAC4 at 6 hours while cisplatin already diminished its protein levels (Figure 2C). Finally, we used a xenografted tumor model to establish that the inhibition of HDAC4 expression was also observed *in vivo* (Figure 2D).

### The organoruthenium complex RDC11 presents a distinct transcriptomic signature

The ability of RDC11 to interact directly with histones and its impact on histone post-translational modification through epigenetic regulation suggested that RDC11 might alter a broad range of signaling pathways. Hence, to identify without bias, RDC11 deregulated signaling pathways we performed a transcriptome analysis. In addition, to identify similarities and specificities in the gene regulations caused by ruthenium-based complexes versus platinum-based complexes, we treated cancer U87 cells at the IC_50_ values for RDC11 (2 μM) and cisplatin (3 μM) (Supplementary Figure S3A). We chose these cells because the IC_50_ of the two drugs are close allowing us to identify gene regulations under similar treatment conditions. We also tested at two time points, 6 h and 24 h, in order to identify early and later gene regulation events and their possible temporal and functional relationships. For the identification of regulated genes, we used Affymetrix hugene10stv1 arrays and each condition (Control: Ct; RDC11 6 h: R6; RDC11 24 h: R24; cisplatin 6 h: C6; cisplatin 24 h, C24) was performed in triplicate.

**Figure 3:**
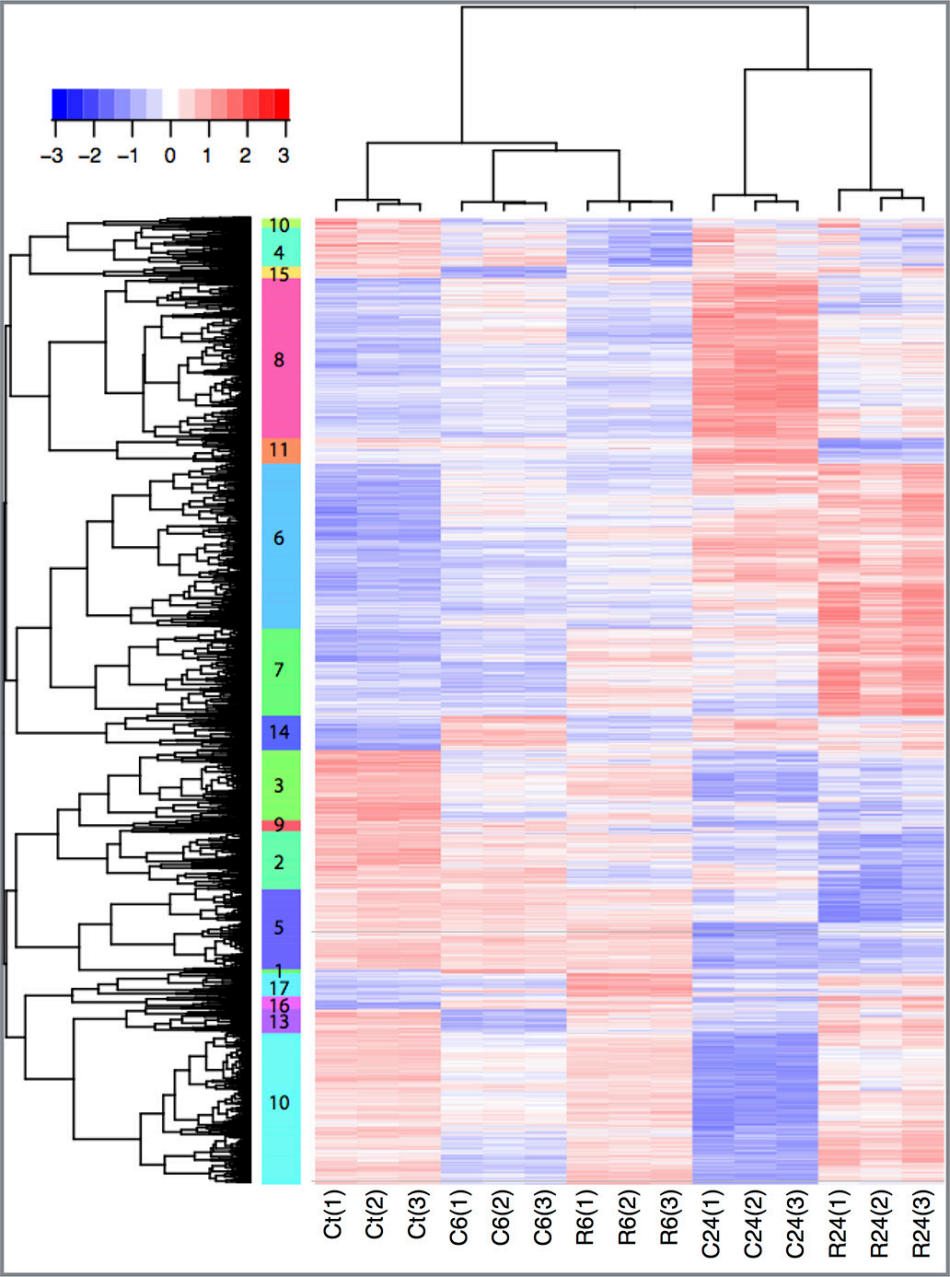
Hierarchical clustering Clustering of expression of 4540 probe-set significantly regulated in at least one treated group compared to control. Rows represent gene expression and columns biological samples. Gene expression levels are represented as scaled expression values (row Z-score from −3 to +3). Blue: low expression, Red: high expression, white: moderate expression. 17 different clusters are detected, indicated by their number and different color next the probe-set tree. C6: Cisplatin exposure for 6 hrs; C24: Cisplatin exposure for 24 hrs; R6: Ruthenium exposure for 6 hrs; R24: Ruthenium exposure for 24 hrs.

**Figure 4:**
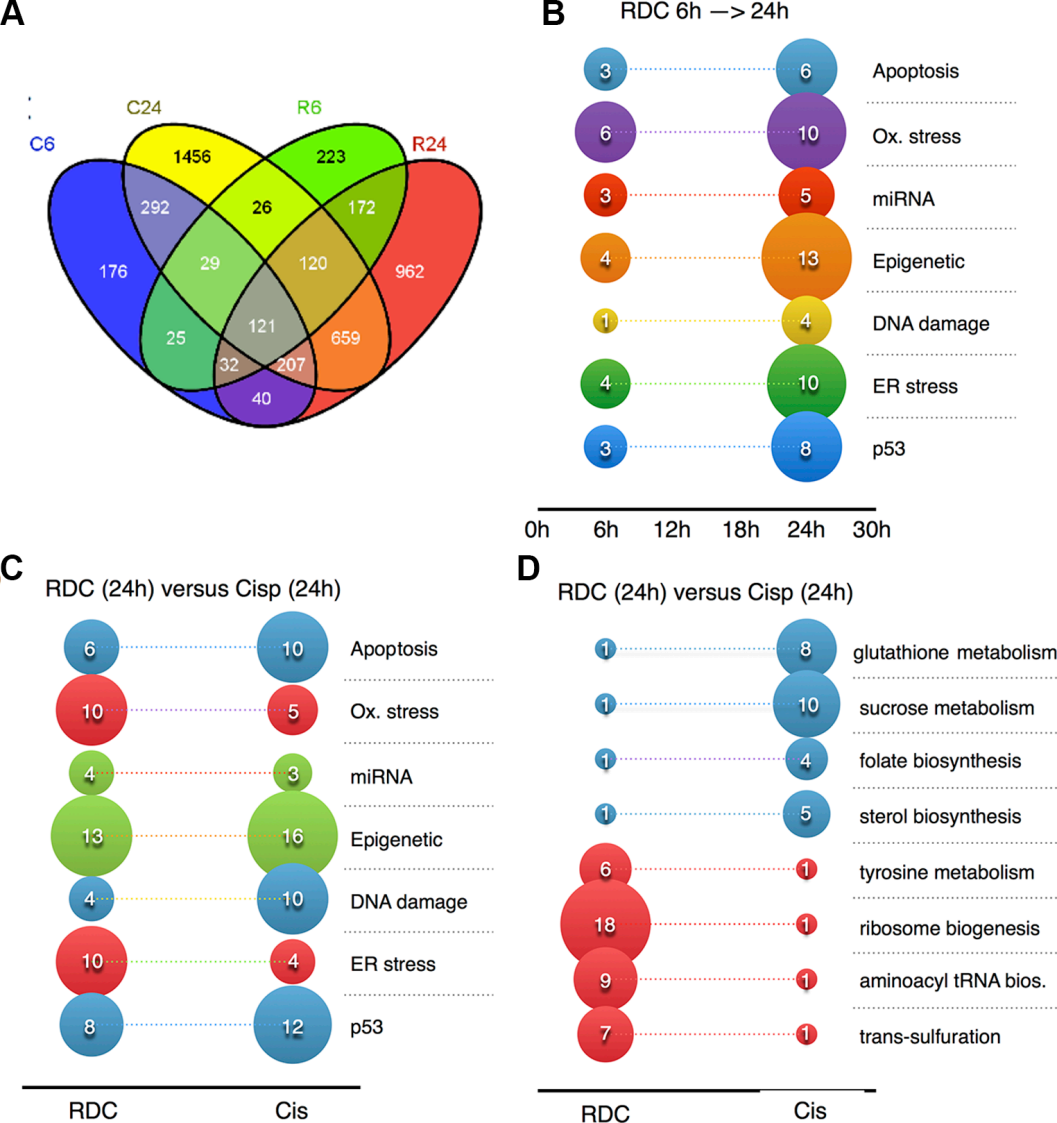
Signaling pathways and mechanisms regulated by cisplatin or RDC11 (**A**) Venn diagram. Significantly deregulated probe sets between control and exposed conditions are compared to highlight genes shared or specific to each group. C6: Cisplatin exposure for 6 hrs; C24: Cisplatin exposure for 24 hrs; R6: Ruthenium exposure for 6 hrs; R24: Ruthenium exposure for 24 hrs. (**B**, **C**, **D**) Graphs represents number of genes in the indicated pathways that are regulated by RDC11 at 6 hours or 24 hours (B) or by RDC11 and cisplatin at 24 hours (C, D). Microarray data were analyzed using AltAnalysis and R bioinformatics tools to identify in KEGG, Gene Ontology, miRNA, transcription factors databanks, the signaling pathways and mechanisms corresponding to the mis-regulated genes.

Principal component analysis of the normalized data using RMA and probe-level linear models validated that the data are reproducible (Supplementary Figures S1A, S2, S3B). To detect differentially-expressed genes, we performed the comparative analysis between control and treated groups 6 hours and 24 hours after exposure. 4540 probe sets with fold change ≥ ± 1.5 and adjusted *p*-value ≤ 0.05 were considered as significantly regulated in at least one comparative analysis. To find groups of genes with similar expression patterns across the different conditions and thus potentially involved in the same regulatory pathways, we performed hierarchical clustering analysis on the set of 4540 de-regulated probe-set (Figure 3). Analysis of the 4540 genes revealed that 922 probe-sets are de-regulated by cisplatin and 748 probe-sets by RDC11 6 hours after exposure (Figure 4A, Supplementary Figure S1B). After 24 hours, 2910 and 2314 probe-sets are regulated by cisplatin and RDC11 respectively (Figure 4A). 22% to 28% (*n* = 207) of the probe-sets are regulated both by RDC11 and cisplatin 6 hours after treatment. These numbers increase to 38% to 48% (*n* = 1107) by 24 hours (Figure 4A).

In our hands, several relevant groups of genes were deregulated. Differences in gene regulation are already detected 6 hours after exposure. For instance the cluster 10, 4 and 15 are made of genes downregulated after 6 hours of cisplatin treatment compared to control. Overall, the effect on gene regulation is greater after 24 hours both in term of genes regulated and differences in gene expression levels (Figure 3). One group of genes (cluster 6, *n* = 776) is characterized by low/moderate gene expression levels 6 hours after treatment with RDC11 or cisplatin, and high expression levels after 24 hours treatment. Gene set enrichment analysis revealed that this cluster is highly enriched for genes with functions in apoptosis (FDR < 10^–5^) such as p21, IRF1, NFKBIB, I-kB, ATF-3, PP2A regulatory, Caspase-7, C/EBP zeta, NF-kB, PPP2R5A, suggesting that apoptosis is induced by both chemicals after 24 hours. In addition to this common set of genes, clustering revealed a specific group of genes induced either by RDC11 or cisplatin. For instance the cluster 8 (*n* = 753), which is characterized by high expression levels 24 hours after cisplatin treatment, is enriched for genes in “apoptosis and survival Apoptotic Activin A” signaling (FDR < 10^–3^) such as Activin A, ActRIIA, Bcl-XL, p53, H-Ras, SHIP, c-Fos, while this pathway is not significantly regulated after 24 h RDC11 treatment (R24). Cluster 7 (*n* = 409) is another example highlighting intrinsic differences in the transcriptional changes induced by RDC11 and cisplatin with a group of genes highly expressed in R24 and with moderate or low expression levels in other conditions. Some effects of the cisplatin are mediated by specific down regulation of a set of 710 genes (cluster 10) enriched for function in cell adhesion via Ephrin signaling (FDR < 10^–5^) such Ephrin-A, Tiam1, FAP-1, Ephrin-A5, Fyn, GRB10, Intersectin, VAV-2, NCK2 (Grb4), FAK1 (Supplementary Table T1). Clustering into the different groups was confirmed by soft clustering (Supplementary Figure S1C) [32]. This, identifyies again a group of genes, soft cluster A, showing a moderate decrease in their expression at 6 hours, which is further downregulated at 24 hours of treatment with cisplatin or RDC11, respectively. Another example for the different effects of cisplatin and RDC11 on a group of genes is soft-cluster B, where cisplatin shows only a very low/moderate effect on their expression, whereas this group of genes is strongly upregulated by RDC11 at 24 hours of treatment. Together, these data suggest that cisplatin and RDC11 share some mechanism activating apoptosis, but overall the mechanism of actions of these two drugs is distinct. Indeed, although the absolute number of genes regulated in common by both chemicals reached a maximum of 48% 24 hours after treatment (Figure 4A), the levels of regulation as well as the early and late responses are different, making the transcriptional imprint unique for each chemical in the conditions tested here.

In order to assess a possible relative generalization of the findings to other cancer cell types and to validate some of the regulations observed using the arrays, we performed RT-qPCR analyses on U87 cells, HCT116 colon cancer cells and AGS gastric cancer cells. For this, several genes were chosen randomly and a representative set is shown in Supplementary Figures S3D and S4. Results were compared to the data obtained by the microarray analyses. In each cell line, drugs were applied at their IC_50_ value for 6 or 24 hours. RT-qPCR experiments showed a good correlation with the results of the arrays for U87 or HCT116 cells. However, the correlation was not as strong for AGS cells, indicating that some of the mechanisms might be cell line-specific.

### The organoruthenium complex RDC11 modulates pathways distinct from cisplatin

As indicated above, gene set enrichment analyses of different databases identified pathways commonly or differently affected by RDC11 and cisplatin. Figure 4B shows the number of genes identified by gene set enrichment analyses that are upregulated in RDC11 treated cells for the indicated pathway between 6 hours and 24 hours when compared to control conditions. As already seen in the clustering analyses (Figure 3), the overall regulations intensified with time, such as genes that are involved in apoptosis (i.e. APAF1, Caspase 3). In correlation with apoptosis-related genes, several genes involved in selected pro-apoptotic pathways, such as p53 target genes, ER stress-related and oxidative stress-related genes are induced over time. Importantly, the expression of several genes of these pathways is already induced at 6 hours. In addition, genes involved in other cellular processes, such as several DNA damage-related genes, a few miRNAs, and multiple genes encoding enzymes involved in epigenetic control are upregulated over time by RDC11.

Interestingly, when we compared the number of genes involved in these pathways and present at 24 hours in RDC11 or cisplatin-treated cells, several differences can be identified (Figure 4C). For example, p53-, DNA damage- and apoptosis-related genes are more present in cisplatin-treated cells. Inversely, ER stress- and oxidative stress-related genes are more frequent in RDC11-treated cells. Note that the number of miRNAs and epigenetic-related genes present in RDC11- and cisplatin-treated cells is similar. Strikingly, gene set enrichment analyses revealed that several cellular metabolic processes were very selectively regulated by either RDC11 or cisplatin (Figure 4D). For example, trans-sulfuration and aminoacyl tRNA synthetase are selectively induced by RDC11. In contrast, genes involved in sterol biosynthesis and sucrose metabolism are preferentially regulated in cisplatin-treated cells.

Altogether the gene set enrichment analyses indicated that RDC11 and cisplatin present each a preference in signaling pathways they regulate. These results clearly demonstrate that cisplatin and the ruthenium-based complex RDC11 have a different mode of action and point to some potential mechanisms that may account for the cytotoxicity of RDC11.

### Cisplatin is a more potent inducer of the p53 pathway than the organoruthenium complex RDC11

**Figure 5:**
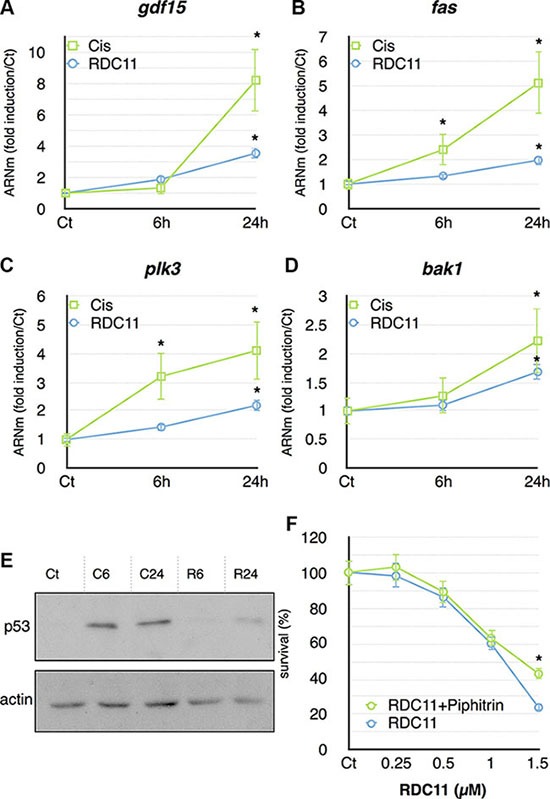
mRNA levels of gdf15 (A), fas (B), plk3 (C) and bak1 (D) were assayed in cancer cells by RT-qPCR Curves are means of fold induction versus the control (Ct) with SD (*n* = 3). *:*p* < 0.01. (**E**) Proteins were extracted from cells treated with RDC11 (R6, R24) or cisplatin (C6, C24) for the indicated time. Western blot analysis revealed p53 and actin expression. (**F**) Survival curve of AGS cells treated with RDC11 and pifithrin-α (10 μM). Cells were treated for 48 hours in 96-well plates and their survival was evaluated by MTT assay (*n* = 8).

To further validate some of the compound specific signatures identified by the gene set enrichment analyses, we performed RT-qPCR to measure the expression of p53 target genes in HCT116. Cells were treated for 6 hours and 24 hours with cisplatin or RDC11 at their IC_50_. *Gdf15*, *fas*, *bak1* and *plk3* were all induced by cisplatin and RDC11 at 24 hours (Figure 5A, 5B, 5C, 5D). However, the induction by cisplatin was more pronounced and occurred often already at 6 hours of treatment. We then monitored the protein levels for p53 under the same experimental conditions. P53 proteins were significantly more abundant in cisplatin-treated cells compared to RDC11-treated cells (Figure 5E). In addition, increased p53 protein expression was already seen at 6 hours in cisplatin-treated cells. Although only weakly induced by RDC11, we assessed the importance of p53 in RDC11 biological activity using pifithrin, which is considered to be a p53 inhibitor [33]. Survival of cancer cells was assessed using MTT assays. Pifithrin did not block the biological activity of RDC11 except at doses above the IC_75_ values (75% of the maximal effect of RDC11, Figure 5F), while it had a significant effect on cisplatin cytotoxicity (Supplementary Figure 3E). The results indicated that RDC11 is less capable of inducing p53 signaling than cisplatin, but p53 may still play a role in the biological activity of RDC11.

### RDC11 is a more potent inducer of the ER pathway than cisplatin

**Figure 6:**
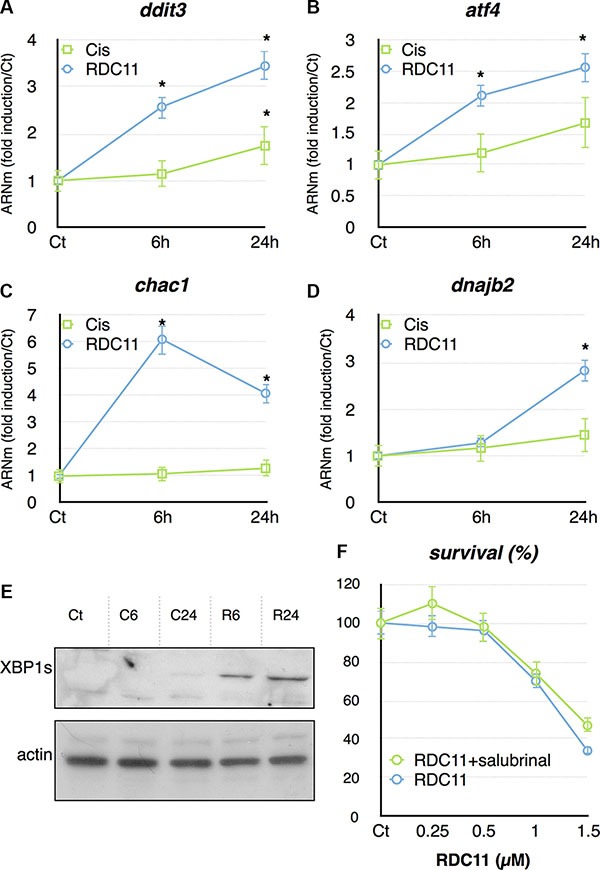
mRNA levels of ddit3 (A), atf4 (B), chac1 (C) and dnajb2 (D) were assayed in cancer cells by RT-qPCR Curves are means of fold induction versus the control (Ct) with SD (*n* = 3). *:*p* < 0.01. (**E**) Proteins were extracted from HCT116 treated with RDC11 (R6, R24) or cisplatin (C6, C24) for the indicated time. Western blot analysis revealed XBP1s and actin expression. (**F**) Survival curve of AGS cells treated with RDC11 and salubrinal (10 μM). Cells were treated for 48 hours in 96-well plates and their survival was evaluated by MTT assay (*n* = 8). Bars are means of fold induction versus the control (Ct) with SD (*n* = 3). *:*p* < 0.01.

We also focused on the ER stress pathway, which represented a more selective signature for RDC11. RT-qPCR showed that several genes, *ditt3*, *atf4*, *chac1*, *dnajb2*, involved in the ER stress pathway, were preferentially induced by RDC11 (Figure 6A, 6B, 6C, 6D). Some of these genes were also induced by cisplatin, but to a significantly lesser extent. To further document the induction of the ER stress pathway we analyzed the production of the spliced form of XBP1 (XBP1s). XBP1s is generated from IRE1 that is itself activated by the ER stress pathway. Figure 6E shows that RDC11 strongly induced the production of XBP1s while cisplatin had no or little effect. To assess whether eif-2a, a component of the ER stress pathway, is essential in the biological activity of RDC11 we used salubrinal, a known inhibitor of eif-2a [34]. Cell survival was assessed by a MTT assay and revealed that salubrinal did not significantly alter the cytotoxicity of RDC11 (Figure 6F). These results showed that although RDC11 stimulates preferentially the expression of components of the ER stress pathway, the activity of eif-2a is not required for RDC11 cytotoxicity.

### An HDAC inhibitor antagonizes RDC11 activity but synergizes with cisplatin in gastric cancer cells

The gene set enrichment analyses also showed a strong deregulation of genes involved in epigenetic mechanisms (Figure 4B and 4C) confirming our previous observations on the effect of cisplatin and RDC11 on HDAC gene expression (Figure 2B, 2C, 2D).

**Figure 7:**
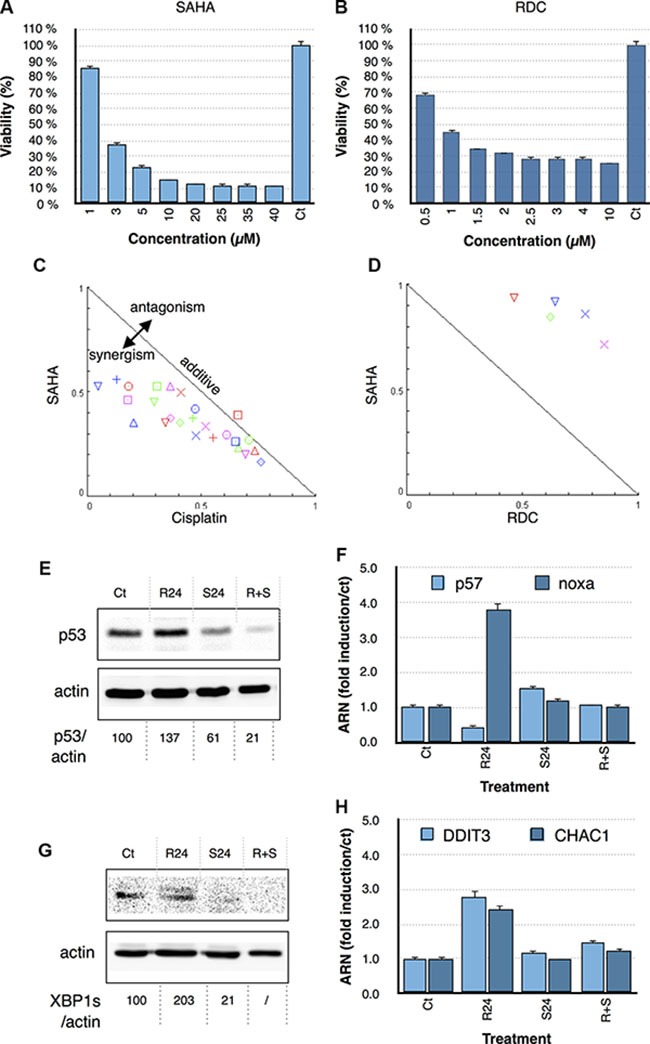
Survival curve of AGS cells treated with SAHA (A) and RDC11 (B) Cells were treated for 48 hours in 96-well plates and their survival was evaluated by MTT assay (*n* = 8). Bars are means of fold induction versus the control (Ct) with SD (*n* = 3). *p < 0.01. Isobologram analyses and combinatory index representations of combinatory treatments between cisplatin with SAHA (**C**) and RDC11 with SAHA (**D**). Doses at IC_75_, IC_60_, IC_50_, IC_30_, IC_25_ were combined and the results were analyzed with the algorithm of Chu and al. using the CompuSyn software. (**E**, **G**) Proteins were extracted from cancer cells treated with RDC11 (R24) or SAHA (S24) or RDC11 and SAHA (R = S) after 24 hours of treatment. Western blot analysis revealed p53 (E) and XBP1s (G) and actin expression. Quantifications are indicated below as measured by Pixi imager. (**F**, **H)**. mRNA levels of *p53* (A), *noxa* (B), *ddit3* (C) and *chac1* (D) were assayed in cancer cells by RT-qPCR. Graphs are means of fold induction versus the control (Ct) with SD (*n* = 3). *p < 0.01. (E) Proteins were extracted from cells treated with RDC11 (R24) or SAHA (S24) or RDC11 and SAHA (R+S) for the 24 hours.

To further assess the importance of HDAC regulation in cisplatin and RDC11 biological activity in cancer cells, we used the HDAC inhibitor SAHA (Vorinistat). MTT assays were first performed to establish the dose response curve of RDC11, cisplatin and SAHA in AGS cells (Figure 7A, 7B). In a second step, combination treatments were performed by combining the two drugs at specific concentrations (i.e. IC_25_, IC_50_) accordingly to the isobologram protocol [35]. The results were then analyzed using isobologram statistical analyses. This analysis, performed with different combinations of HDAC inhibitor and RDC11, showed an antagonistic effect of the HDAC inhibitor on the cytotoxicity of RDC11 (Figure 7D). In contrast, the HDAC inhibitor synergized with cisplatin's cytotoxicity (Figure 7C). These results showed again a significant difference between the mode of action of RDC11 and cisplatin. In particular, this suggests that HDAC activity might be required for RDC11 cytotoxicity but not for cisplatin.

To understand how SAHA might impact on RDC11 biological activity, we analyzed the p53 and the UPR pathway. As previously observed, RDC11 induced p53 and XBP1s protein levels as well as their target genes (Figure 7E, 7F, 7G, 7H). Interestingly, co-treatment with SAHA significantly diminished the impact of RDC11 on both signaling pathways. These series of results indicate that the epigenetic regulation controlled by RDC11 plays a role in RDC11 biological activity and changes the signaling pathways controlled by RDC11.

## DISCUSSION

Nowadays, the precise identification of the mode of action of a drug destined for therapeutic purpose is crucial. In oncology, it allows the selection of the group of patients that have the best chance of responding to the treatment in an “a la carte” (personalized) therapeutic approach in which the mode of action of the drug matches the genetic profile of specific tumors. It also provides markers to monitor the activity of the drugs during the therapy and point to the direction of a possible combination therapy by combining the compound with other therapeutic molecules. In addition, the identification of the direct target(s) is a clear advantage in developing a rational optimization process aiming at an improvement of the therapeutic activity. The general development of ruthenium-based anticancer compounds toward clinical use has been clearly handicapped by the lack of consensus about their mode of action and the long-lasting misconception that ruthenium-based compounds represent a succedaneum of platinum-based compounds by acting via the same targets. To answer this complex problem we investigated the possible non-DNA direct targets of a ruthenium based complex (RDC11) and performed an unbiased transcriptomic approach to assess the repercussion of these interactions on cellular signaling pathways key for the control of cell survival. The results of this study provided a comprehensive view on how RDC11 targets histones and illustrate its cellular consequences. In addition, the unbiased and global transcriptomic approach presented in this study provides an unique perspective on the respective characteristics of the organoruthenium compound RDC11 and cisplatin by providing information at two levels: i) a statistical evaluation of the difference and the similarities between the genes regulated by both drugs, ii) a list of novel genes, signaling pathways and metabolic processes regulated specifically by RDC11 that represents a source of information for pathways potentially regulated by other ruthenium-based compounds. Finally, this study illustrates also how such information may provide hints for developing novel combination treatments. In this present case, we focused on histone deacetylase (HDAC) regulation.

### An organoruthenium compound interacts with histones

Our study shows that the organoruthenium compound RDC11 interacts with several histones (H3, H2A and H2B) in cells (Figure 1C) and *in vitro* with purified histones (Figure 1D). This finding represents one of the very few examples of a non-DNA target for ruthenium complexes that has been validated using cellular extract. The ability of RDC11 to interact with purified histones suggests strongly that the interaction is direct. In addition, the fact that the interaction is still observed after several washes or in a polyacrylamide gel also indicates that the interaction is relatively stable. It remains to be established more precisely how the compound interacts with histones, such as whether it involves specific interactions and how it affects the proteins at the molecular and structural level. The migration pattern of the RDC11/histone complex shows a tendency of increased trimeric forms, suggesting that the interaction between RDC11 and histone may favor formation of histone complexes (Figure 1D). This is also supported by the fact that using different protein extraction buffers, we observed that treatment of the cells with RDC11 leads to a decrease in the more soluble fraction of histones in the cells (Figure 1E). This set of results supports a preceding finding that a ruthenium complex of piano stool structure called READ-C interacts with histones [36]. In addition, our study is the first demonstration of an organoruthenium compounds that interact with histone in cells and it also shows that ruthenium compounds have an impact on the post-translational modifications of histones. These modifications are part of complex and essential epigenetic mechanisms that are essential and complex control of gene expression [31].

### An organoruthenium compound impacts on epigenetic regulations

It is expected that the interaction between a ruthenium compound and histones may alter the complexes in which histones are involved: DNA/histones and histones/epigenetic enzymes modifying histones, Indeed, we observed that, in cells treated with RDC11 histones have a different solubility and post-translational modification pattern. For instance, histone H3 levels are lower in the more soluble fraction and its acetylation on lysine 9 is increased (Figure 2). Interestingly, the acetylation pattern is different between RDC11 and cisplatin. It remains to be established whether the modification of solubility and acetylation is directly due to the binding of RDC11 to histone H3. Addressing this specific point represents a complex technical challenge.

Similarly, understanding precisely the relationship between the interaction of RDC11/histones and the transcriptional regulation affecting the epigenetic enzymes will be also challenging. Indeed, if RDC11 alters the expression of HDAC and demethylases (Figures 2B–2DB, 4B–4CB), we still need to assess whether these are adaptation/compensation mechanisms of the chromatin to the structural perturbation due to RDC11 binding, or whether it is a consequence of the modification of chromatin structure leading to different accessibility to transcription factors. Indeed, RDC11 treated cells show a change in the expression of several HDACs and other epigenetic modifiers. These changes are different to those induced by cisplatin. For instance, HDAC4 is strongly repressed by cisplatin whereas RDC11 induced significantly HDAC9. These changes already happen 6 hours after treatment suggesting that they participate into the gene expression regulation observed in cancer cells. The fact that these changes are also observed *in vivo* (Figure 2D) further support their importance for RDC11 and cisplatin's biological and anticancer activity. To elucidate the impact on gene expression of these epigenetic modifications induced by RDC11 and/or cisplatin transcriptomic data were analyzed.

### Specificities in RDC11 and cisplatin transcriptomic profiles

Bio-statistical analyses of the microarray data showed significant differences in the number and quality of the genes regulated by RDC11 or cisplatin. Among the 4540 genes that we identified to be regulated, less than a third were common to RDC11 and cisplatin (Figures 3, 4). About 40% were specifically regulated by cisplatin and a third by RDC11. This difference is even more marked at 6 hours post-treatment, as only 1.5% of the regulated genes are common between cisplatin and RDC11-treated cells. The clustering analyses further highlighted the existence of 17 gene clusters that show significant variation of the expression profile over time between RDC11 and cisplatin after treatment (Figures 3, 4). The significant differences in the transcriptome controlled by RDC11 and cisplatin indicate that both compounds should trigger different signaling pathways, likely reflecting distinctive mode of actions.

A more detailed analysis of the signaling pathways or cellular mechanisms that are controlled by the regulated genes confirmed the existence of common and distinctive effects between RDC11 and cisplatin. Apoptosis, DNA damage, p53, epigenetic, miRNA, ER stress and oxidative stress were amongst the mechanisms that were regulated by both drugs (Figures 4, 5, 6). However, the number and the intensity of activation of the genes included in those pathways were often significantly different between RDC11 and cisplatin. For example, cisplatin induces a high number of genes involved in DNA damage, p53, and apoptosis, while RDC11 favors oxidative- and ER stress-related genes. The differences between both drugs are even more accentuated on specific metabolic pathways (Figure 4D). For example, RDC11 specifically regulates ribosome biogenesis, while cisplatin favors sucrose metabolism.

### Respective contribution of the p53 and ER stress pathway in RDC11 activity

Some of these specificities were previously documented by our group, such as the preferential activation of the p53 pathway by cisplatin or the ER stress pathway by RDC11 in glioblastoma cells [26, 27, 30]. Here, we further extended these observations by showing that these specificities are common to cancer cells of different origin, such as colon cancer cells (HCT116) or gastric cancer cells (AGS). In addition, we extended the observation to an additional pool of genes and to the protein level for a marker of the ER stress pathway (Figures 5, 6, 7). Altogether, these novel observations validate the preferential activation of the ER stress pathway by RDC11 in different cancer cell lines. With the endoplasmic reticulum being also the place of protein translation, it is interesting to note that RDC11 triggered a ribosomal biogenesis response along with the ER stress pathway (Figure 4).

Interestingly, the preferential activation of the p53 pathway by cisplatin correlates with a preferential activation of DNA damage response. Hence, compared to RDC11, cisplatin appears to induce a coherent DNA-damage/p53 pathway response, which is also consistent with the preferential binding of cisplatin to DNA as compared to RDC11 [26, 27, 30]. We previously described this pathway to contribute to the neurotoxic activity of cisplatin [37, 38].

The exact contribution of the p53 pathway and the ER stress pathways in RDC11 activity have already been investigated previously in glioblastoma cells. Using a dominant inhibitor and p53-/- cell lines, we previously showed that p53 is not absolutely necessary for RDC11 cytotoxicity [26, 27]. We confirmed here these results by showing the RDC11 induces less the p53 pathways compared to cisplatin and that a known inhibitor of p53 (pifithrin) does not drastically alter the activity of RDC11. We also inhibited eIE2a, a component of the ER stress pathway, with salubrinal, which did not alter RDC11 cytotoxicity. This result apparently contradicts previous data showing that the ER stress transcription factor CHOP is partly necessary for RDC11's full cytotoxicity [27]. This apparent contradiction highlights the complexity of the ER stress pathway and might be explained by the fact that the activation of eIE2a by PERK represents only a part of the mechanisms triggered by the ER stress pathway [39]. For example, CHOP can be induced by other mechanisms related to the ER stress pathway, such as ATF6.

### Respective role of epigenetic regulators in RDC11 and cisplatin cytotoxicity

Amongst the mechanisms that were regulated differently by RDC11 and cisplatin, several genes encoding for histones and histone-modifying enzymes were included. These genes and in particular HDACs, are playing a critical role in various biological process and represent interesting therapeutic targets [40]. Our study showed that whereas EZH2 was induced by both RDC11 and cisplatin, HDAC4 was preferentially repressed by cisplatin. This repression of HDAC4 by cisplatin was also observed *in vivo* in fragments of human colon cancer xenografted in nude mice (Figure 2D). In addition, we observed that these gene regulations impact on the acetylation of histone H3 at lysine 9 in a time- and drug-dependent manner. In particular, 6 hours after treatment, RDC11 represses H3 acetylation while cisplatin already induces it. Such differential drug response correlates with a profound difference in the impact of HDAC inhibitors on RDC11 and cisplatin's biological activity. Indeed, a known HDAC inhibitor, SAHA, had a synergistic anticancer activity with cisplatin in AGS human gastric cancer cells as previously observed by others with cancer cell lines of a different origin [41]. In contrast, we demonstrate here that an HDAC inhibitor (SAHA) inhibits RDC11 biological activity as revealed by the antagonistic effect observed in the isobologram analysis. This result suggests that, in contrast to cisplatin, RDC11's biological activity might require the activity of some HDACs by modifying either the histones or some of their other targets. From a more therapeutic point of view, this study indicates that RDC11 is not a good candidate to be used in combinatory therapy with general HDAC inhibitors for it might depend on the expression and/or activity of HDACs in the tumor. It also confirms the potential advantage of combining HDAC inhibitors with platinum-based therapies in gastric cancers.

From a more mechanistic point of view, we also show that the combination of RDC11 with SAHA impact on the signaling pathways regulated by RDC11. The induction of p53 and Noxa is almost annihilated by SAHA when combined with RDC11. Similarly, SAHA reduced XBP1's? signaling (Figure 7). These changes in signaling may account for the differential biological activity of RDC11 in the presence of SAHA.

Overall this study demonstrates that RDC11 and cisplatin have different modes of action and trigger the regulation of different signaling pathways. Whereas both agents lead to regulation of pro-apoptotic effectors and cell cycle inhibitors, the intermediate mechanisms are quite different. These differences have profound consequences as they will define how we might use RDC11 on tumors with specific signatures (i.e. p53 mutated or not) or with specific other therapeutic molecules. It remains for us to establish how these findings can be generalized to other ruthenium-based compounds or even other metal-based compounds. Our previous studies, as well as those of others, showing that ruthenium- or osmium-based compounds with different ligands trigger similar regulation of p53 and ER-stress pathways, suggest that the findings of this study might point out potential signaling pathways targeted by other metal-based compounds [26] [19, 20, 42, 43].

## MATERIALS AND METHODS

### Chemicals and purified proteins

Cisplatin, pifithrin, SAHA and salubrinal were purchased from Sigma and used as received. RDC11 was synthesized and purified as previously described [18]. DNA-free purified histones were purchased from New England Biolabs.

### Cell cultures

U87, AGS and HCT116 cells were obtained from ATCC. Cells were manipulated and cultured in DMEM with 10% FCS (Dominique Dutcher^TM^) and 1% Penicillin + Streptomycin (Sigma) at 37°C with 5% CO_2_ atmosphere as previously described [44]. All experiments were conducted by comparing the treatments with cells treated with buffer without the active compounds (cisplatin, ruthenium complex or SAHA).

### Cell survival assay

2000 cells were seeded per well in 96-well microplates (Falcon Multiwell), 48 h prior to any treatment. Cisplatin and RDC11 were applied for 48 h in fresh medium. MTT assay was performed as previously described by replacing the medium with fresh medium supplemented with 5 mg/L MTT (Sigma) for 1 h [45]. Cells were lysed in isopropanol with 0.04 N HCl. Measurements were performed at 550 nm.

### Quantitative PCR

Cultured cells were lysed with 1ml of TRIzol (Invitrogen) per 10×10^6^ cells and RNA extracted according to the manufacturer's instructions. RNA samples were ethanol-precipitated twice and 1 μg was used for reverse transcription (High-Capacity cDNA Reverse Transcription Kit, Applied Biosystems). RNA quality was assessed by qPCR was performed using 2 ng/μl cDNA (RNA equivalent) according to the manufacturer's instructions (SYBR Green PCR Master Mix, Applied Biosystems) and with 400 μM of each primer (Supplemental data, Table 1). The relative expression was calculated using the ΔΔCt method. Expression levels were normalized using average of 18 S or TBP.

**Table 1:** List of primers used in qPCR

Primers	Gene
Cggtgctggacatatgagac	HDAC1 left
Tggtccaaagtattcaaagtagtca	HDAC1 right
Gcctgaagaactctaagccaga	HDAC9 left
Ggaacttctgacttcacatccac	HDAC9 right
Gcactgcataagcagatgaga	HDAC8 left
Tttccgtcgcaatcgtaata	HDAC8 right
Gtggtagagctggtcttcaagg	HDAC4 left
Gaccacagcaaagccattc	HDAC4 right
Ttgttggcggaagcgtgtaaaatc	EZH2 left
Tccctagtcccgcgcaatgagc	EZH2 right
Agcggcaagatgtacttcca	Ephb2 left
Ggagccgatgatgagtgg	Ephb2 right
Hs01121172_m1 (TaqMan GEA)	p21
GGTCAGTCCCTCCAACAACA	ATF4 left
CTATACCCAACAGGGCATCC	ATF4 right
Aaggcactgagcgtatcatgt	DDIT3 C Left
Tgaagatacacttccttcttgaacac	DDIT3 C rigth
Agatcatgagggctgcactt	CHAC1 left
Gtggcaatggcctcttca	CHAC 1right
Cttttgcagagctctttgatga	DNAJ2 left
Aggaggagaaatcggagtgc	DNAJ2 right
Cacgatggagcgtcttgtc	p57 left
Gctcagctcctcgtggtc	p57 right
Gtggatttcctgggcaag	noxa left
Tcatggttcgctcctggt	noxa right

### Statistical analysis

Statistical analyses were performed using a one-way ANOVA test followed by a Tukey pos *t*-test allowing a comparison between all the conditions. * Indicate statistical differences in graphs. Statistical Analyses were performed using Prism (GraphPad software).

### Western blotting

Cells or tissue were lysed with LB (125mM Tris-HCl pH 6.7, NaCl 150 mM, NP40 0.5%, 10% Glycerol). Proteins (20 μg) were denatured and deposited directly (75 μg of proteins) onto a SDS-PAGE gel. Western blotting was performed using antibodies raised against p53 (rabbit anti-p53, FL-393, Santa Cruz, CA), XBP1s (Santa Cruz, CA), EZH2 (Cell Signaling), H3 (Cell Signaling), H3K9 (Abcam) and HDAC4 (Biolegend). Secondary antibodies (anti-rabbit, anti-mouse: Sigma, MA) were incubated at 1:1000. Loading was controlled with actin (rabbit anti- β-actin, Sigma, 1:4000) [46].

## SUPPLEMENTARY MATERIALS METHODS FIGURES


