# Supplementary material for: A ruthenium anticancer compound interacts with histones and impacts differently on epigenetic and death pathways compared to cisplatin
Source: Oncotarget. 2016 Nov 30;8(2):2568–84. doi: 10.18632/oncotarget.13711 (PMC5356824; doi:10.18632/oncotarget.13711)
Supplement: Supplementary file 1 [file oncotarget-08-2568-s001.pdf]

A ruthenium anticancer compound interacts with histones and impacts differently on epigenetic and death pathways compared to cisplatin

Supplementary Materials

Statistical analysis of microarray data

Data obtained from Affymetrix hugene10stv1 arrays (http://www.ncbi.nlm.nih.gov / geo / query / acc.cgi?acc=GSE66493) were analyzed in R version 3.0.1. Raw data were first extracted from the CEL files and visualized using the package affyPLM to detect potential hybridization artifacts. Probeset annotations were retrieved from Ensembl (GRCh37.p10 release 69) with Biomart (BMC Genomics 2009, 10:22 doi: 10.1186 / 1471-2164-10-22) for 32325 probe-set corresponding to 25489 mapped genes. Normalization was performed either with RMA model from the affy package (Bioinformatics. 2004 Feb 12; 20(3):307-15.) or with probe-level linear models (affyPLM) (Bioinformatics. 2004; 20:307–315). The normalization methods were compared by plotting ROC curves using the respective adjusted p-values values as predictor. Differential expression analysis was performed with the limma package (BMC Genomics. 2006 Oct 9; 7:252. Clustering was performed on a selection of 4540 probsets showing at least one condition with $|FC| \geq 1.5$ and adjusted p-value ≤ 0.05. Hierarchical clustering was carried out with Pearson's correlation and the complete-linkage method. Clusters were defined by 1.5 times the height of the longest branch. Soft clustering was performed with the package Mfuzz using the parameters $c = 8$ and $m = 1.6$ (30). Gene Set enrichment analysis was carried out with MetaCore (Thomson Reuters) using human annotations, *p*-values ≤ 0.05 were considered as significant. Pathway analysis and transcription factor target analysis were performed with AltAnalysis.

RDC11-matrix synthesis

The RDC11 containing support used for affinity chromatography was obtained by coordinating the hypogel-400-CO(-5-amino-1,10-phenanthroline) resin to a coordinatively-unsaturated ruthenium derivative containing a cycloruthenated phenyl pyridine ligand.

Synthesis of the phenanthroline containing support

Commercially available (Interchim) Hypogel-400-COOH (1 g, 0.3 mmol) and hydroxybenzotriazole (HOBt) 0.268 g, 2mmol) were added in DMF/CH2Cl2 (2:1, 15 mL). To this solution EDCI.HCl (1-Ethyl-3-(3-dimethylaminopropyl)carbodiimide.HCl) (0.379 g, 2 mmol) and diisopropylethylamine (60 µL (microL), 4 mmol) were added. After 30 min. stirring, 5-amino-1,10-phenanthroline (0.129 g, 0.66 mmol) suspended in DMF was added and the reaction mixture was stirred for 24 h at room temperature. The resulting solid support was filtered and washed consecutively with DMF/CH2Cl2 (5/5 mL), CH2Cl2 (10 mL) and Et2O (10 mL). After drying in high vacuum the modified resin was used as such for the next step.

Synthesis of the RDC11 containing support

The modified support obtained above (0.5 g, 1.0 eq.) was suspended in dry CH2Cl2 (10 mL) to which was added [Ru(2-phenyl-2'-pyridine)(MeCN)4]PF6 (50.028 mg, 1 eq.) ref. The reaction mixture was stirred for 24 h at room temperature. The black solid support was filtered and washed consecutively with DMF/ CH2Cl2, CH2Cl2 and Et2O. Drying this solid under high vacuum afforded the required RDC11-containing support.

Digestion

In gel digestion

Stained protein bands were excised prior to destaining, in-gel reduction and alkylation of proteins, which were performed using a MassPREP Station (Waters, Manchester, UK). Briefly, destaining was done by three washes in a mixture containing 25 mM NH4HCO3:CH3CN (1:1, v:v) followed by 30 min of dehydration in acetonitrile at 60°C for 5 min. Cysteine residues were reduced by 50 µL of 10 mM dithiothreitol, 25 mM NH4HCO3 at 57°C for 30 min and alkylated by 50 µL of 55 mM iodoacetamide, 25 mM NH4HCO3 for 30 min. After washing with 50 µL of 25 mM NH4HCO3, dehydration was done with acetonitrile during 15 min. Proteins were cleaved in-gel using 40 µL of 12.5 ng/µL of modified porcine trypsin (Promega, Madison, WI, USA) in 25 mM NH4HCO3 at 37°C for 4 hours. Tryptic peptides were first extracted using a 60% acetonitrile solution containing 0.5% formic acid, then secondly with a 100% acetonitrile solution.

Liquid digestion

Sample were dried and solubilized in 200 µl of 8 Murea in 100 mM NH4HCO3 buffer. Each sample was reduced for 1 h at 60 °C by adding dithiothreitol to a final concentration of 10 mM. Alkylation was performed by adding iodoacetamide to a final concentration of 40 mM, 1 h at room temperature. To carry out the digestion in an optimal way, the sample was diluted to 1 M urea. An overnight digestion was performed by adding trypsin in a 1:50 enzyme to protein ratio. All samples were desalted on Sep-Pak C18 cartridges.

LC-MS/MS and data analysis

NanoLC-MS/MS analyses were performed on two systems: i/ a nanoACQUITY Ultra-Performance-LC system (UPLC) coupled to a SYNAPT HDMS Q-TOF mass spectrometer equipped with a nano-electrospray source (Waters), ii/ an Agilent 1100 series nanoLC-Chip/MS system (Agilent Technologies, Palo Alto, USA) coupled to an ion trap (amaZon, Bruker Daltonics, Bremen, Germany).

NanoACQUITY Ultra-Performance-LC system coupled to a SYNAPT HDMS Q-TOF

nanoACQUITY UPLC system and SYNAPT HDMS Q-TOF mass spectrometer were controlled by MassLynx v4.1 (SCN 566, Waters). The solvent system consisted of 0.1% formic acid in water (solvent A) and 0.1% formic acid in ACN (solvent B). Peptides were injected and first trapped during 3 min on a precolumn (Symmetry C18, 20 mm × 0.18 mm, 5 µm particle size, Waters) at a flow rate of 5 µL/min with 99% A, then eluted at 45°C on a separation column (ACQUITY UPLC® BEH130 C18, 200 mm × 75 µm, 1.7 µm particle size, Waters) at a flow rate of 400 nL/min using a 35 min linear gradient from 1 to 40% B and followed by 5 min at 65% B. The mass spectrometer was operating in positive mode with the following settings: source temperature was set to 80°C, cone gas flow was 30l/h, cone voltage was 40V and the nanoelectrospray voltage was optimized to 3.5 kV. Mass calibration of the TOF was achieved using phosphoric acid (H3PO4) on the 50-2000 m/z range. Online correction of this calibration was done using product ions derived from the [Glu1]-fibrinopeptide B (GFP) as lock-mass compounds. The ion (M + 2H)2 + at m/z 785.8426 was used to calibrate MS data and the fragment ion (M + H) + at m/z 684.3469 to calibrate MS/MS data.

Spectra were acquired by automatic switching between MS and MS/MS modes. This was done in the mass range of 250–1500 m/z (MS, 0.5 sec) and 50–2000 m/z (MS/MS, 0.7sec). The most abundant peptide ions (the 3 most intense with a threshold of 60 counts/sec), preferably with a charge of 2 to 4, were selected from each MS spectrum for further isolation and CID (Collision Induced Dissociation) fragmentation using argon as collision gas. Ions were excluded after acquisition of one MS/MS spectrum and exclusion was released after 0.6 min. NanoLC-MS/MS raw data generated were respectively converted into « .pkl » peaklists with PLGS 2.3 (Waters).

Agilent 1100 series nanoLC-Chip/MS system coupled to an amaZon ion trap

This system was fully controlled by HyStar 3.2 (BrukerDaltonics). The chip contained a Zorbax 300SB-C18 column (43 mm×75 µm, 5 µm particle size) and a Zorbax 300SB-C18 enrichment column (40 nL, 5 µm particle size). The solvent system consisted of 2% acetonitrile, 0.1% formic acid in water (solvent A) and 2% water, 0.1% formic acid in acetonitrile (solvent B).

3 µl of each sample were loaded into the enrichment column at a flow rate set to 3.75 µL/min with solvent A. Elution was performed at a flow rate of 300 nL/min with a 8–40% linear gradient (solvent B) in 30 min (in gel) or 45/90 min (liquid sample, short/long gradient), followed by a 4 min stage at 70% of solvent B before reconditioning the column at 8% of solvent B.

MS spectrums were acquired with the following settings: source temperature was set to 135°C while cone gas flow was at 3 L/min. The nanoelectrospray voltage was optimized to −1850 V. The MS spectra were acquired in the positive ion mode on the mass range 250 to 1500 m/z using the standard enhanced resolution at a scan rate of 8.1 m/z/s. The Ion Charge Control was fixed at 200000 with a maximum accumulation time of 200 ms. For tandem MS experiments, the system was operated with automatic switching between MS and MS/MS modes. Fragmentation was performed using argon as the collision gas. The 6 most abundant peptides were selected on each MS spectrum for further isolation and fragmentation with a preference for doubly charged ions (absolute threshold of 5000, relative threshold 5%). Ions were excluded after the acquisition of 1 MS/MS spectra and the exclusion was released after 0.3 min. The Smart Parameters Setting option was used for the selected precursor ions. The MS/MS spectra were acquired on the mass range 100 to 2000 m/z. The Ion Charge Control was fixed at 300000 and 2 scans were averaged to obtain a MS/MS spectrum.

The system was fully controlled by HyStar 3.2 (BrukerDaltonics). Mass data collected during the nanoLC-MS/MS analyses were processed and converted into .mgf files using the DataAnalysis 3.3 Build 146 software (Bruker Daltonics).

Protein identification

Peaklists (.pkl and .mgf) were searched using a local Mascot server (version 2. 2. 0, MatrixScience,

London, UK) against a combined target-decoy protein database containing protein sequences of Homo sapiens (taxonomy 9606) derived from UniprotKB (created 2015-10–30, 84456 entries) and common contaminants such as human keratins and trypsin. The database was created using an in-house database generation toolbox (Carapito and al. 2014). Database searches were performed using the following settings: trypsin was specified as enzyme and up to one missed cleavage by trypsin and three variable modifications (oxidation of methionine, carbamidomethylation of cysteine and acetylation of protein N-termini) were considered. Mass tolerances on precursor and fragment ions were set to 20 ppm and 0.2 Da, respectively for system 1 and 50 ppm and 0.2 Da for system 2. Mass tolerances on precursor and fragment ions were set to 25 ppm and 0.07 Da for SYNAPT and 250 ppm and 0.5 Da for Amazon.

Identifications were validated with the in house developed Proline software (http://proline.profiproteomics.fr/) using the following validation criteria: target/decoy validation applying a 1% FDR at protein level. Carapito and al. 2014

Proteomics. 2014 May;14(9):1014-9. doi: 10.1002/pmic.201300415. Epub 2014 Mar 12. MSDA, a proteomics software suite for in-depth Mass Spectrometry Data Analysis using grid computing. Carapito C, Burel A, Guterl P, Walter A, Varrier F, Bertile F, Van Dorsselaer A.

REFERENCES

1. Fernandez S, Pfeffer M, Ritleng V, Sirlin C. Organometallics. 1999; 18:2390–2394.

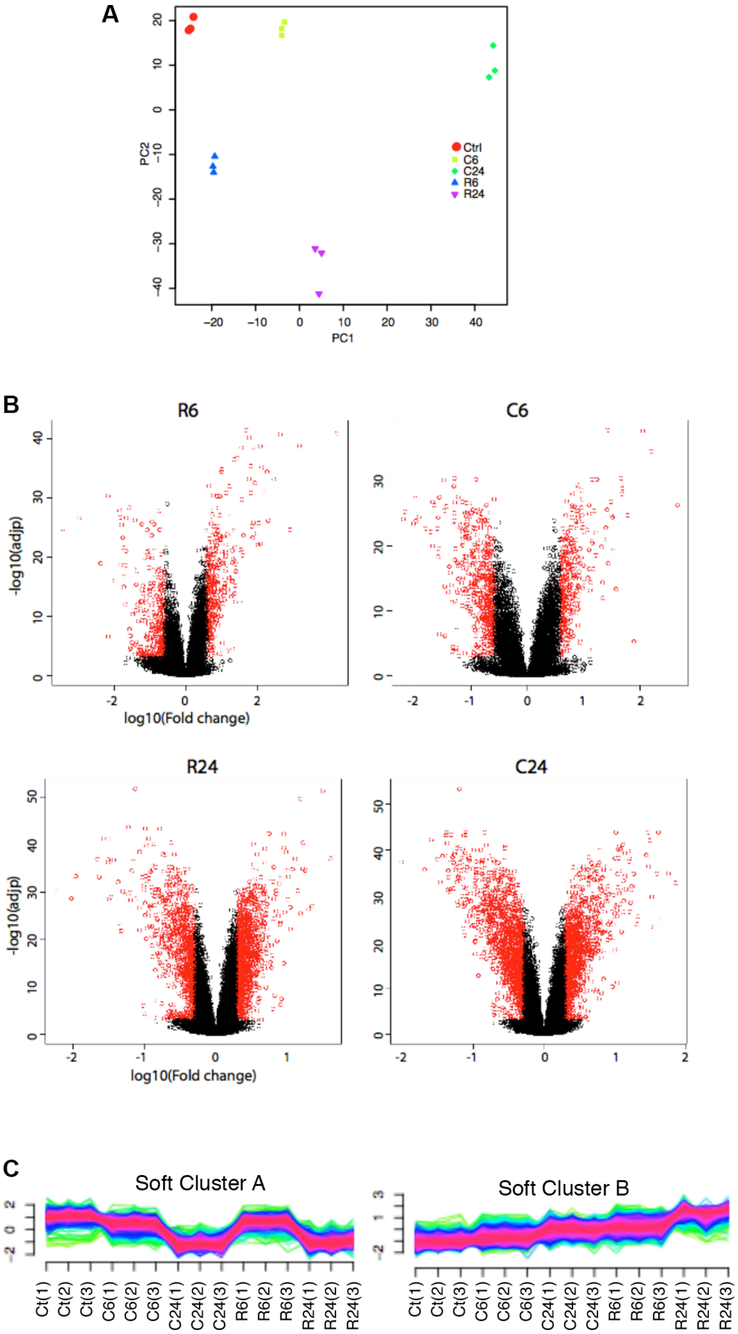

Supplementary Figure S1: (**A**) Principal component analysis. Plotting of two principal components from RMA normalized data using probe probe-level linear models. Biological replicates for the 5 different conditions cluster together and no outliers are detected within the dataset. (**B**) Vulcano plots. Plotting of the log10 of the fold change (x-axis) in function of the -log10 of the adjusted p values (y-axis). Unexposed cells are used as reference. Significant differential expression in microarray data are shown in red for the four comparative analysis using the threshold: $|FC| \geq 1.5$ and adjusted p-value ≤ 0.05. (**C**) Soft clustering of the normalized microarray data. The set of 4540 mis-regulated genes was subjected to soft clustering to find gene families sharing similar expression patterns. Genes belonging to the same cluster, ie. with high membership, are colored in red. Normalized and scaled expression levels are indicated on y-axis. Samples names are indicated below each graph.

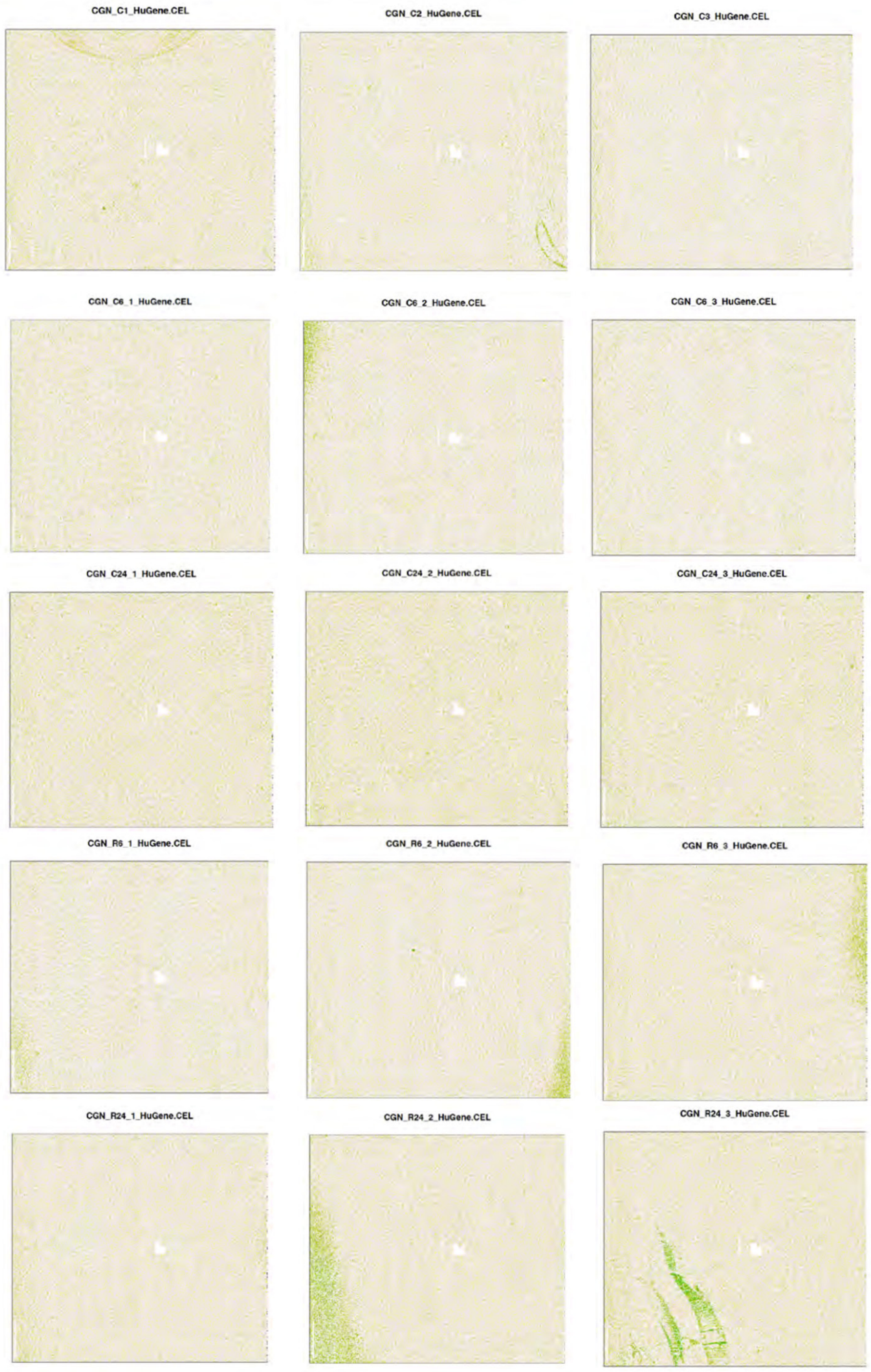

Supplementary Figure S2: Pictures of the microarray hybridization results.

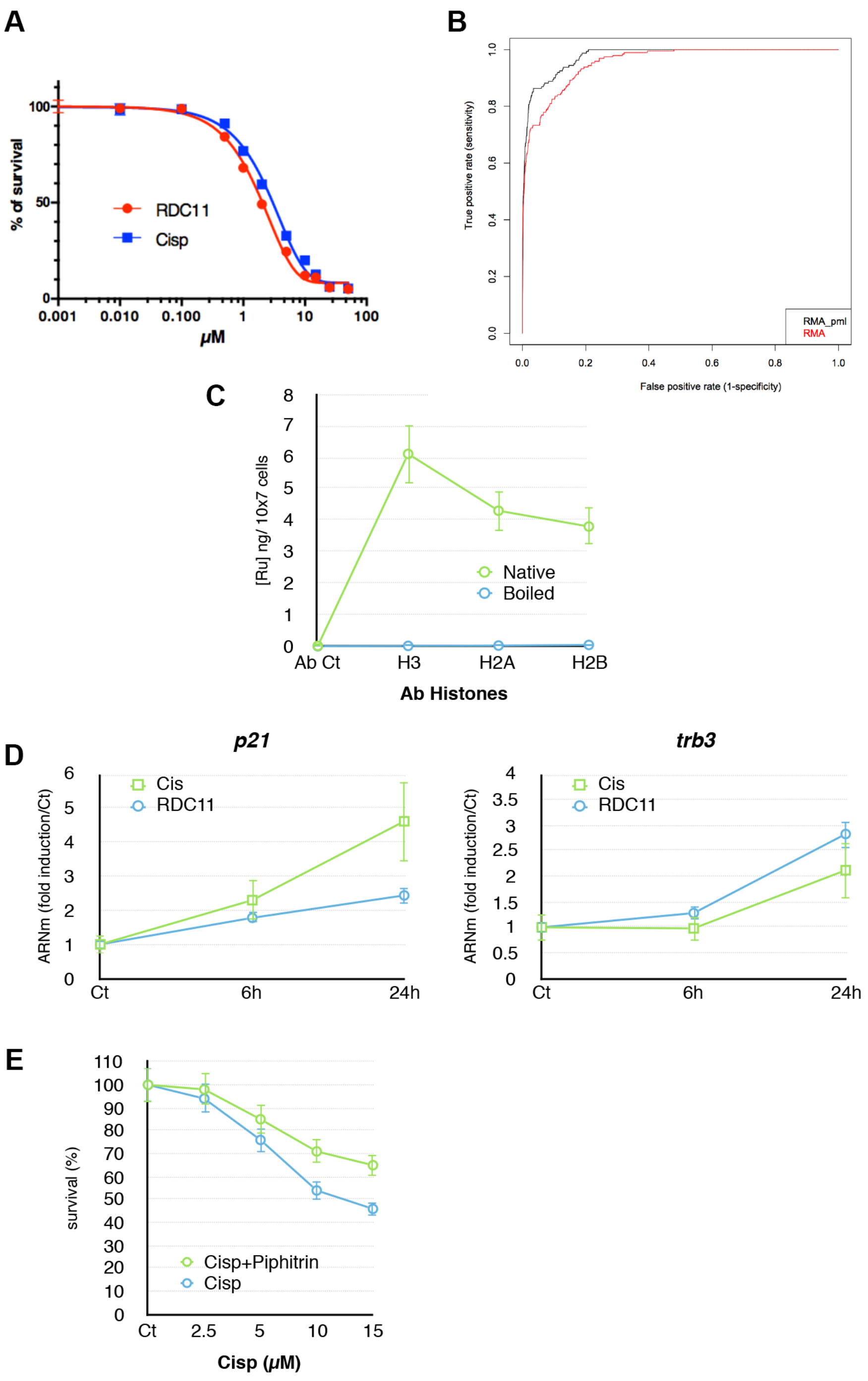

Supplementary Figure S3: (**A**) MTT of U87 cells treated with RDC11 and Cisplatin for 48 hours**.** (**B**) Graph representing microarray statistical analysis. (**C**) Plot of cellular [Ru] accumulation on each histone when treated at a fixed concentration of 5 µM. Ru amount determined by measurement with ICP-MS on precipitates of histones using corresponding antibodies. Mean ± SD of three independent experiments. (**D**) mRNA levels of genes found disregulated in the microarraus: p21 and trb3 were assayed by RT-qPCR. Curves are means of fold induction versus the control (Ct) with SD ($n = 3$). *$p < 0.01$. (**E**) Survival curves of AGS cells treated with cisplatin in absence or in presence of pifithrin ($n = 8$). *$p < 0.01$.

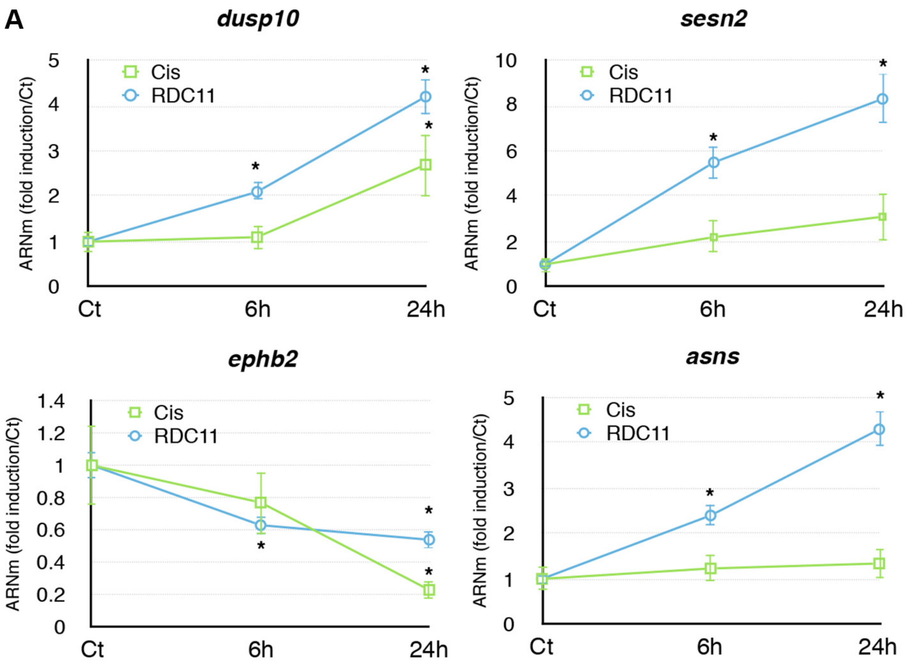

B

		Array		RT-qPCR		RT-qPCR AGS		RT-qPCR HCT116	
		6h	24h	6h	24h	6h	24h	6h	24h
dusp10	*RDC*	+2.5	+4	+2	+4.3	-2.1	-1.2	+4.1	+7.1
	Cis	+1.5	+2.6	+1.1	+2.8	-2.2	-3.1	+1	+1
sesn2	*RDC*	+3.5	+5	+5.8	+8.1	+2	+2.4	+12.2	+7.1
	Cis	+1.7	+2.7	+2.1	+3.3	+1	-1.3	+3.3	+2.1
ephb2	*RDC*	+1	-2.8	–1.6	–1.8	1	1.5	-1.4	-5.2
	Cis	+1	-4.8	–1.3	–3.8	1	-5.6	-1.4	-5.1
asns	*RDC*	+5.2	+5.2	+2.4	+4.3	1	1.5	-1.4	-5.3
	Cis	+3.6	5.7	+1.2	+1.3	-4.9	-2.3	1	-2.1

Supplementary Figure S4: mRNA levels of genes found disregulated in the microarraus. (**A**) dusp10, sesn2, eph2, asnas were assayed in U87 (A, B RT-qPCR) colon cancer HCT116 (**B**) and gastric cancer AGS (B) cells by RT-qPCR. Curves are means of fold induction versus the control (Ct) with SD ($n = 3$). *$p < 0.01$. In B, number indicate average of fold changes.

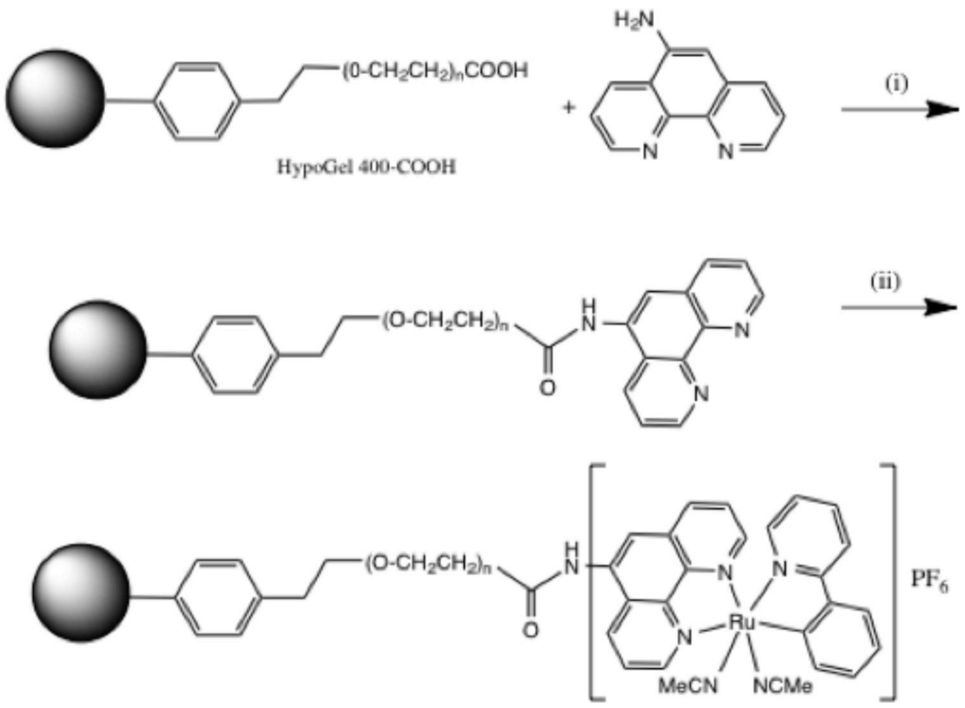

Supplementary Figure S5: Supplementary experimental procedures.
